# Dynamic Analysis of Deep Water Highway Tunnel under Ocean Current

**DOI:** 10.1155/2022/9551792

**Published:** 2022-03-31

**Authors:** Li Fang, Hong Li, Bin Li

**Affiliations:** School of Transportation and Logistics Engineering, Wuhan University of Technology, Wuhan 430063, China

## Abstract

Comprehensively comparing the merits and demerits of the existing means of transportation across the water, a new underwater transportation structure for crossing the wide water area, named as “deep water highway tunnel” (hereinafter called “DWHT”), is proposed. The characteristics of flow field around the typical section of DWHT at different flow velocities are investigated, which can provide reference for the values of hydrodynamic coefficient at high Reynolds number. The vibration modes and natural by the sound-solid coupling method. In addition, considering the factors of fluid-structure coupling, the dynamic response of displacement and internal force is analyzed based on CFD for the weak parts of the structure. The results show that the deepening of water and the increase of flow will significantly increase the flow field pressure and structure stress, and when the span (or width-span ratio) of the tunnel body extends beyond a certain range, the dynamic characteristics and dynamic response rules of the structure will change.

## 1. Introduction

The core problem of underwater tunnel construction is to ensure the stability and safety of the tunnel structure system in harsh marine environment, accurately predict the hydrodynamic response of the structure system, and obtain the technical parameters such as motion, deformation, and stress, which are the primary scientific and technological problems faced by the design, construction, and safe service. At present, scholars have made some achievements in the research about the underwater structure under wave and flow load.

Svein et al. [1] proposed an alternative method for random dynamic response analysis of SFT under wave loads. Hiroshi et al. [2] conducted two-dimensional model tests for the wave environment of Hokkaido, and the dynamic characteristics of SFT are studied by boundary element numerical simulation. Di Pilato et al. [3] chose Messina channel SFT as the research object, considering the coupling vibration effect of pipe body and anchor cable, and studied its nonlinear time-history response under the action of steady flow and wind-induced waves, as well as the local dynamic behavior of anchor cable under the action of earthquake. Tariverdilo et al. [4] established two-dimensional and three-dimensional models to simulate the dynamic response of suspended tunnel under moving load, considering the inertia effect of fluid. The results showed that the response values of the two models and their differences gradually decreased with the increase of tension leg stiffness. Seo et al. [5] carried out physical model tests in two-dimensional wave tanks for accurately estimating the hydrodynamic force and proposed a pendulum model used to describe the motion of SFT under single vertical mooring.

Mai et al. [6] took the lead in exploring the static and dynamic response of SFT under the combined action of wave and flow loads and focused on the analysis of the dynamic response of vortex-induced vibration. Wang et al. [7] and Luo et al. [8] respectively used RNG model and LES modal to analyze the pressure distribution characteristics around different sections and the variation rules of hydrodynamic parameters. Li et al. [9] studied the effects of random irregular waves on the underwater tunnel in a two-dimensional wave flume experiment. Fan and Yuan [10] explored the influence of different design parameters on the dynamic response of the underwater tunnel and also studied on the vortex-induced resonance characteristics based on the differential equation of vibration including nonlinear factors; the results show that choosing a feasible span length was the most effective measure to control the vortex-induced resonance response. Yang et al. [11] researched on vehicle partial load effect, with a tunnel tube with a total span of 1 km as the research object, based on Hamilton principle.

Compared with the existing transportation means across water, this paper proposes a new underwater transportation structure, named as “deep water highway tunnel.” As a matter of experience about underwater tunnels, several researches on the dynamic analysis of the DWHT in the ocean current are conducted, including a series of numerical simulations based on fluid-structure coupling methods to investigate the characteristics of flow field around typical sections, as well to explore the mechanism and relevant influence factor of natural vibration characteristics and dynamic response of deep water highway tunnel.

## 2. A Concept: Deep Water Highway Tunnel

Researches on new means of transportation across water areas have been gradually emerging. In terms of the alternative modes to cross broad water areas, the main transportation modes include ferry, bridge, and underwater tunnel. In recent years, underwater tunnel has attracted attention for its unique advantages.

Ferry, as the most traditional modes of cross-sea transportation mode, has numerous advantages of mature technology, low construction cost, large transportation volume, and so on. However, it is easy to be affected by climate, and the fuel consumption is large, which makes ferry not suitable for the fast pace of modern urban transportation. As for the cross-sea bridge, it is convenient for vehicles to pass, with low operating costs and strong capacity. Nevertheless, there are also various problems such as geographical limitations, vulnerability to adverse weather, high cost, and difficulties of underwater construction.

Underwater tunnels are divided into undersea tunnels, immersed tunnels, and submerged floating tunnels according to the depth of the pipeline axis. The surrounding rock environment of undersea tunnel is considerably complex, leading to a challenge to carry out geological investigations. Furthermore, the long construction period, the staggering cost of the undersea tunnels. and frequent accidents such as landslides and water gushing are actually hard nuts to crack. Immersed tunnel has higher requirements on the flatness of submarine topography and limits the maximum buried depth. The construction technology of foundation groove excavation and foundation treatment is complicated, which will also cause certain damage to the marine ecological environment. Submerged floating tunnel (i.e., SFT) [12, 13] is a new type of underwater technology, although there are no completed projects of SFT at present. Especially when anchor cable is used as the support system, the cost is high, underwater installation is complex, and vortex-induced resonance is easily induced which severely limits the horizontal spacing of anchor system, and numerous other technical difficulties are still urgently needed to be settled.

**Figure 1:**
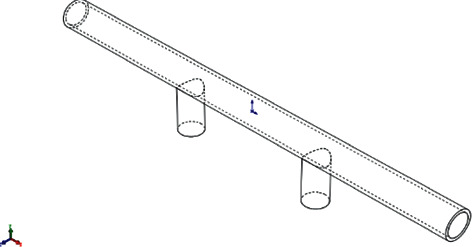
Basic structure diagram of DWHT.

The conception of deep water highway tunnel is based on the structural model of the cross-sea bridge and the submerged floating tunnel. As shown in Figure 1, its basic structure is composed of a completely closed tunnel pipe for carrying traffic loads and a fixed support system embedded in the seabed in the form of piers and columns.

The whole body is in a certain depth under water. While the superstructure is built in water, the huge buoyancy of water can balance part of the dead weight of the structure. As meantime, the form of pier column as the supporting system in the substructure shows its own superiority. The pier column supporting system not only has stronger supporting stiffness that can bear more dynamic load but also can provide an omnidirectional restrain, both horizontal and vertical displacement of the upper tunnel pipe body are effectively constrained, which can improve the structural stability of the tunnel body.

In addition, from the perspective of engineering construction, the latest research on fixed supported underwater structures is extensive and abundant, and the relevant technologies and experience are relatively sufficient, which indicates a distinct feasibility of DWHT.

**Figure 2:**
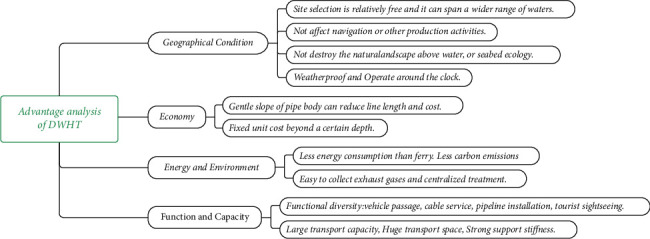
Advantages of deep water highway tunnel.

As a new traffic infrastructure, DWHT has its particularity and limitation, through a comprehensive comparison of different means of transportation across water, DWHT stands out in geographical condition, economy, environmental protection, application, and other aspects, and the specific advantages of DWHT are shown in Figure 2, whereas limited to some environmental factors, as well as the construction technology, and the pier, as a supporting system, will cause the increase of structure weight, therefore the deep water highway tunnel is more suitable for a wide marine area, where the seabed soil is relatively more bearable.

## 3. CFD Theory and Method

### 3.1. Computational Fluid Dynamics Governing Equations

The basic governing equation including the N-S equation of incompressible viscous fluid and the continuity equation are as follows:

(1) $$\begin{array}{c} \left\{\begin{array}{c} \rho \frac{du}{dt}=-\frac{\partial p}{\partial x}+\mu \nabla^{2}u+f_{x},\\ \rho \frac{dv}{dt}=-\frac{\partial p}{\partial y}+\mu \nabla^{2}v+f_{y},\\ \rho \frac{dw}{dt}=-\frac{\partial p}{\partial z}+\mu \nabla^{2}w+f_{z}, \end{array}\right.\\ \frac{\partial u}{\partial x}+\frac{\partial v}{\partial y}+\frac{\partial w}{\partial z}=0, \end{array}$$

where *u*, *v*, *w* are respectively the velocity components in the *X*, *Y,* and *Z* directions; *f*_*x*_, *f*_*y*_, *f*_*z*_ are respectively the external force components of fluid per unit volume in the *X*, *Y,* and *Z* directions.

Among various turbulence models, *SSTk* − *ω* model can obtain more accurate results when dealing with boundary layer simulation and complex flow separation prediction at high Reynolds number, and its governing equation is shown as follows:

(2) $$\begin{array}{c} \frac{\partial }{\partial t}\left(\rho k\right)+\frac{\partial }{\partial x_{i}}\left(\rho ku_{i}\right)=\frac{\partial }{\partial x_{j}}\left(\Gamma_{k}\frac{\partial k}{\partial x_{j}}\right)+G_{k}-Y_{k}+S_{k},\\ \frac{\partial }{\partial t}\left(\rho \omega \right)+\frac{\partial }{\partial x_{i}}\left(\rho \omega u_{i}\right)=\frac{\partial }{\partial x_{j}}\left(\Gamma_{\omega }\frac{\partial \omega }{\partial x_{j}}\right)+G_{\omega }-Y_{\omega }+D_{\omega }+S_{\omega }, \end{array}$$

where *G*_*k*_ is the kinetic energy of turbulence, *G*_*ω*_ is the generation equation of *ω*, Γ_*k*_, Γ_*ω*_ are respectively the effective diffusion terms of *k* and *ω*, *Y*_*k*_, *Y*_*ω*_ are respectively the divergent item of *k* and *ω*, and *D*_*ω*_ is the quadrature divergence term of *ω*.

### 3.2. Fluid-Structure Coupling Theory

The interaction between seawater and underwater structures is dynamic. On the one hand, the fluid force causes the deformation and movement of the underwater tunnel; on the other hand, the movement of the tunnel structure changes the relative position and velocity between tunnel structure and the fluid, thus changing the fluid force. Therefore, it is necessary to consider the fluid-structure interaction effect.

The acoustic-solid coupling method is used to simulate the fluid-structure interaction in wet modal analysis of the structure. The fluid is regarded as a compressible nonviscous acoustic medium. The fluid action is presented in the form of sound pressure dynamic load on the structure wall, and the sound field and velocity potential have to satisfy the Helmholtz wave equation, which is shown as follows:

(3) $$\begin{array}{c} \frac{1}{C^{2}}\frac{\partial^{2}P}{\partial t^{2}}-\nabla^{2}P=0. \end{array}$$

Acoustic finite element discretization equation is

(4) $$\begin{array}{c} \left[M_{a}\right]\left\{\ddot{p}\right\}+\left[C_{a}\right]\left\{\dot{p}\right\}+\left[K_{a}\right]\left\{p\right\}+\rho \left[R\right]\left\{\ddot{u}\right\}=0, \end{array}$$

where [*M*_*a*_] is the fluid mass matrix, [*C*_*a*_] is the fluid damping matrix, [*K*_*a*_] is the fluid stiffness matrix, [*R*] is the structural-sound field coupling matrix, {*p*} is the sound pressure vector of fluid element node, *ρ* is the fluid density, and {*u*} is the node displacement vector.

Dynamic equation of the structure is

(5) $$\begin{array}{c} \left[M_{s}\right]\left\{\ddot{u}\right\}+\left[C_{s}\right]\left\{\dot{u}\right\}+\left[K_{s}\right]\left\{u\right\}-{\left[R\right]}^{T}\left\{p\right\}=\left\{F_{s}\right\}, \end{array}$$

where [*M*_*s*_] is the structure mass matrix, [*C*_*s*_] is the structure damping matrix, [*K*_*s*_] is the structure stiffness matrix, [*R*]^*T*^ is the transpose of the structure-sound field coupling matrix, and {*F*_*s*_} is the external load vector received by the structure.

Simultaneous equations are obtained by considering the formulas above, and the acoustic-solid coupling finite element equation is shown as follows:

(6) $$\begin{array}{c} \left[M\right]\left\{\ddot{x}\right\}+\left[C\right]\left\{\dot{x}\right\}+\left[K\right]\left\{x\right\}=\left\{F\right\}, \end{array}$$

where $\left[M\right]=\left[\begin{array}{cc} \left[M_{s}\right] & 0\\ \rho \left[R\right] & \left[M_{a}\right] \end{array}\right]$, $\left\{x\right\}=\left\{\begin{array}{c} u\\ p \end{array}\right\}$, $\left\{F\right\}=\left\{\begin{array}{c} F_{s}\\ 0 \end{array}\right\}$, $\left[C\right]=\left[\begin{array}{cc} \left[C_{s}\right] & \text{0}\\ \text{0} & \left[C_{a}\right] \end{array}\right]\;$, and $\left[K\right]=\left[\begin{array}{cc} \left[K_{s}\right] & -{\left[R\right]}^{T}\\ \text{0} & \left[K_{a}\right] \end{array}\right]\;$.

As for the study of dynamic response analysis, the sequential coupling method is used to solve the problem. The most basic principle of fluid-structure coupling is that stress, displacement, and other variables should be equal or conserved on the fluid-structure coupling surface. According to the set order, the results of fluid domain and structure domain are exchanged by the fluid-solid interface, and the fluid control equation and structure control equation are solved in different solvers in turn.

## 4. Numerical Simulation

Dynamic research of DWHT under ocean current is carried out from three aspects: firstly, the analysis of flow field around circular section under different flow velocity. Secondly, the wet modal analysis of typical structures based on the acoustic-solid coupling method. Thirdly, the dynamic response analysis under different parameters.

### 4.1. Modal and Meshing

Ansys Workbench integrates a variety of modules to facilitate the calculation and coupling analysis of flow and solid structures, hence numerical simulations of sea areas are conducted in Fluent module, while Modal, Modal Acoustics, Static Structural, Transient Structural, and other modules were used to analyze the dynamic characteristics and responses of structures.

**Figure 3:**
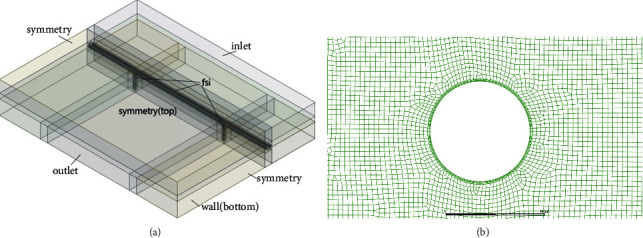
Computing domains and meshing. (a) Compute domain and boundary settings. (b) Local magnification of boundary layer grid.

As the tunnel pipe body and pier body are consolidated as a whole, and the both sides of the midspan are symmetrical, then the following part is taken as the research object, that is, “half span + one span + half span.” In this simplified model, the dynamic response analysis is carried out on the stress condition of pier bottom and the displacement condition of pipe top, ignoring the internal interaction between pipe body and pier body. The form of the typical structure is shown in Figure 3(a).

The cross section width of the pipe body is *D* assumed, and the single-span length is *L*. Making the flow field fully developed and not affecting navigation, the basic size parameters of calculated water area are set as follows: the distance between the inlet and the upstream face is 10*D*, the distance between the outlet and the lee face is 20*D*, the water surface is placed 30 m above the top of the tunnel, the pier is 30 m high, and the axial length along the pipe body is 2 L, whereas the circular section is one of the most common and typical forms; a circular section with a diameter of *D* is selected as the tunnel pipe body.

As shown in Figure 3(a), the top and two sides of the water body were set as symmetry boundaries, the bottom of the water body was set as no-slip wall, and the tunnel pipe surface and pier side surface (i.e., the fluid-solid interface) were set as no-slip wall. Symmetry constraints are set at both ends of the pipe body (remote displacement), and fix support is set at the bottom of the pier.

For the water area, SST*k* − omega model is adopted considering the computational efficiency and the applicability of the model. Moreover, an encrypted boundary layer grid is set on the wall, as shown in Figure 3(b).

### 4.2. Hydrodynamic Coefficient Calculation

Reynolds number is a dimensionless parameter used to characterize fluid flow and reflects the ratio of inertia force and viscosity force. The calculation formula is as follows:

(7) $$\begin{array}{c} R_{e}=\frac{U\;D}{\upsilon }, \end{array}$$

where *υ* is the kinematic viscosity of the fluid, and the value of seawater is 9.738 × 10-7 m^2^/s at 20°C.

Strouhal number is a parameter used to characterize the lift frequency (or vortex discharge frequency) and is related to Reynolds number and junction interface shape. Its definition can be written as follows:

(8) $$\begin{array}{c} S_{t}=\frac{f_{s}V}{D}. \end{array}$$

The drag coefficient and lift coefficient in Fluent are calculated by dividing the lift and drag forces by the reference values of the dynamic pressures. The calculation formulas of drag and lift coefficients are as follows:

(9) $$\begin{array}{c} C_{D}=\frac{{F\;}_{D}}{0.5\rho_{w}Au^{2}},\\ C_{L}=\frac{{F\;}_{D}}{0.5\rho_{w}Au^{2}}, \end{array}$$

where *A* is the area facing of water surface.

The average pressure coefficient calculated in Fluent is

(10) $$\begin{array}{c} C_{p}=\frac{p-p_{\infty }}{0.5\rho_{w}u^{2}}. \end{array}$$

## 5. Results and Analysis

### 5.1. Analysis of Flow Field around Sections

Taking the actual flow field of a certain sea area as the research background, natural condition parameters are as follows: the density of seawater is about 1030 kg/m^3^, the viscosity coefficient is 0.001003 Pa·s, the local coastal surface current velocity is about 0.6–0.8 m/s, and the maximum velocity can reach 2.55∼3.06 m/s, therefore the selected flow rate ranges from 0.5 m/s to 4 m/s in order to make the research more practical, while the Reynolds number is between 5 × 10^6^ and 4 × 10^7^.

The effects of different ocean current velocities on the flow characteristics have been investigated, focusing on the circumferential pressure and lift-drag correlation coefficients of the circular section of the pipe body.

#### 5.1.1. Pressure Field Characteristic

**Figure 4:**
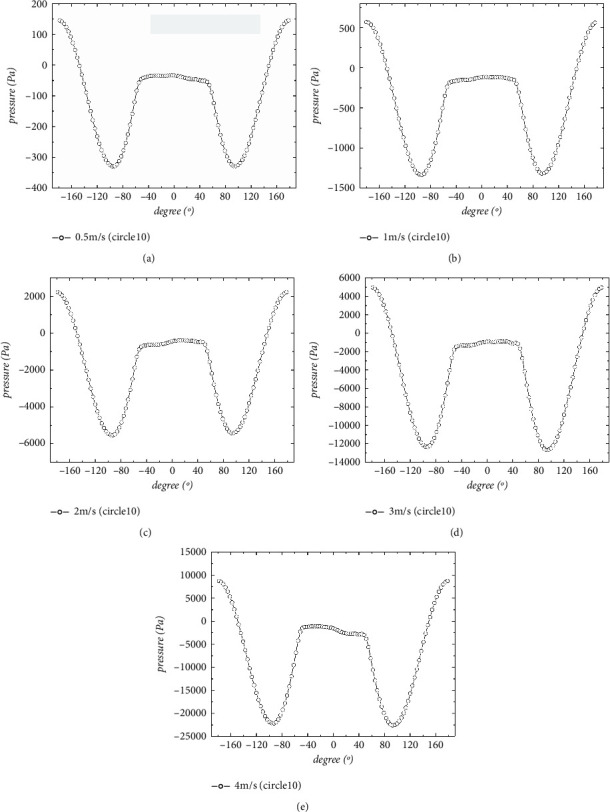
Steady circumferential pressure distribution at different velocities: (a) 0.5 m/s; (b) 1 m/s; (c) 2 m/s; (d) 3 m/s; (e) 4 m/s.

As Figure 4 shows, with the increase of flow velocity, the circumferential pressure around the section increases sharply, and the pressure differential resistance between the front face and back face increases significantly. The pressure distribution curves under different flow rates show the same shape and trend, and the intervals of positive pressure and negative pressure are rarely changed.

**Figure 5:**
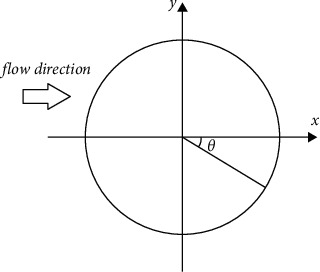
The meaning of *θ* in circumferential pressure diagram.

The meaning of *θ* is illustrated in Figure 5. The maximum negative pressure is reached near the upper and lower vertexes of the cross section, that is, |*θ*| is nearly 90∼100^。^, and the absolute value of the maximum negative pressure is about 2.5∼2.7 times of the maximum positive pressure. While |*θ*| is in the range of 0∼40^。^ on the back flow side, the negative pressure is low and basically unchanged, accessing the stability zone.

**Figure 6:**
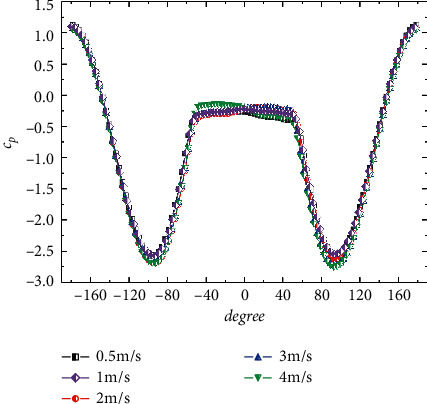
Steady circumferential pressure coefficient at different velocities.

The distribution law of steady pressure around crossing section of tunnel body is consistent at different incoming flow rates. As shown in Figure 6, the distribution curves of the circumferential steady-state pressure coefficients at different flow rates almost coincide. With the velocity increasing, the flow separation point moves slightly closer to the back flow side. The absolute value of the ratio of the maximum negative pressure to the maximum positive pressure increases slightly as the flow rate increases.

#### 5.1.2. Lift and Drag Coefficient Analysis

**Figure 7:**
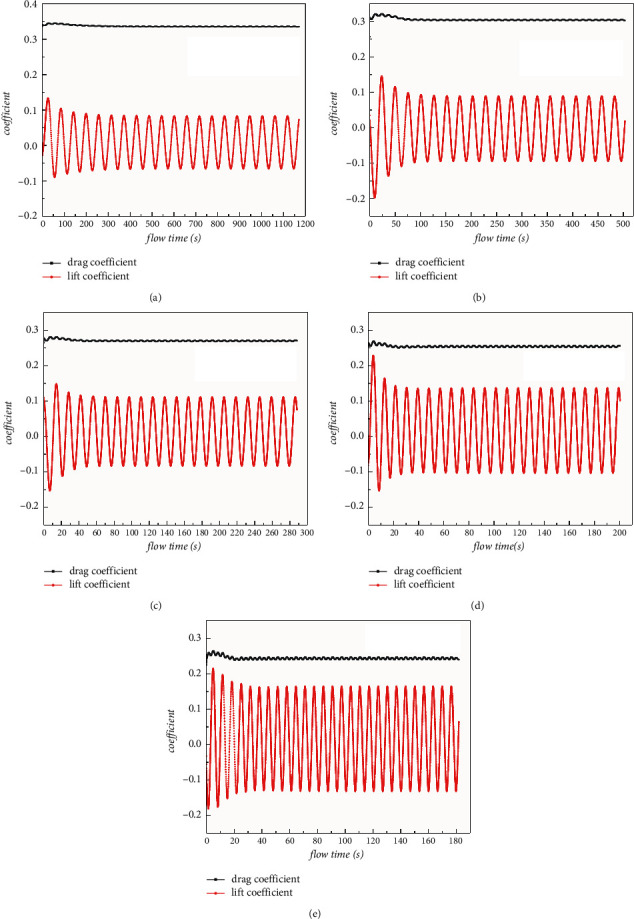
Time-history curves of lift coefficient and drag coefficient at different flow rates: (a) 0.5 m/s; (b) 1 m/s; (c) 2 m/s; (d) 3 m/s; (e) 4 m/s.

The time-history curves of lift coefficients and drag coefficients of the crossing section are shown in Figure 7.

**Figure 8:**
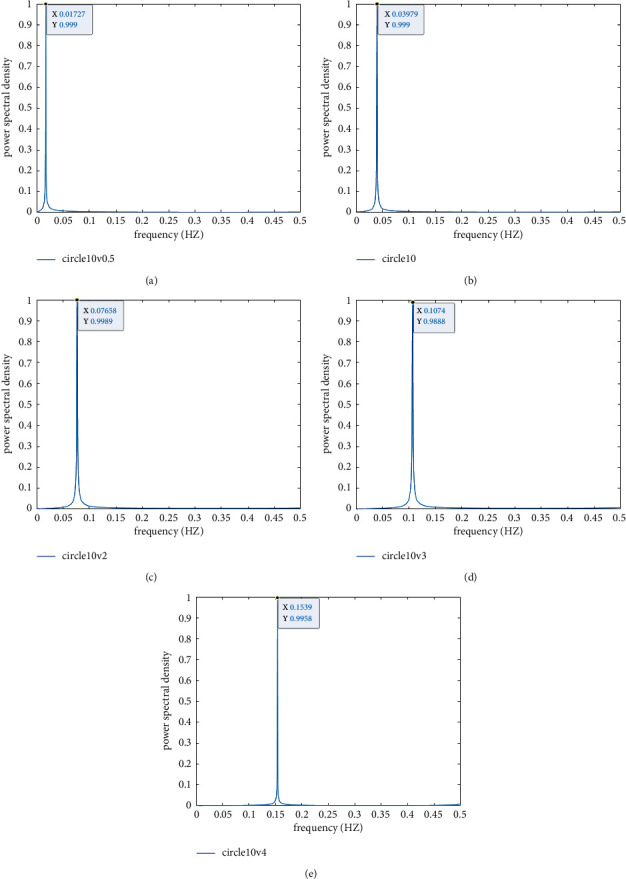
Lift power spectrum at different flow rates: (a) 0.5 m/s; (b) 1 m/s; (c) 2 m/s; (d) 3 m/s; (e) 4 m/s.

The lift power spectrum can be obtained by FFT transformation of the time-history data of the lifting coefficient, and the lift power spectrum at different flow rates is shown in Figure 8.

It can be seen from Figures 7 and 8 that as the flow velocity increases from 0.5 m/s to 4 m/s, the vortex discharge frequency around the cross section gradually increases.

According to the time discrete data of lift and drag coefficients calculated in Fluent, the mean value and the variance value of these data were solved corresponding to steady-state coefficients and fluctuating coefficients.

Table 1 lists the results of the correlation coefficients about lift and drag around cross sections at different flow rates. Combined with the above analysis, it can be seen that the circumferential pressure of the section increases significantly in practice with the increase of the flow velocity, and the lift force and resistance of the structure also increase as well. However, as shown in Table 1, both the fluctuating lift coefficient and the steady-state drag coefficient decrease with the increase of velocity, while the values of steady-state lift coefficient and the fluctuating drag coefficient are small and fluctuate as the velocity changes. In addition, with the increase of flow velocity, the vortex discharge period gradually decreases, and the calculated Strouhal number has a small fluctuation within the process of velocity variation, and the Strouhal number of circular section fluctuates from 0.34 to 0.39.

### 5.2. Analysis of Structural Dynamic Characteristics

The water depth, velocity, and the size of the structure all affect the natural vibration characteristics, and appropriate structural parameters will reduce the possibility of vortex-induced resonance. Referring to the design of section of suspension tunnel [14, 15], the diameter of the circular section is about 10∼25 m, while the length of a single span is about 100∼500 m, hence the size of typical structure of underwater tunnels will be explained in the following sections.

#### 5.2.1. Comparison of Dry and Wet Modes

In the basic example of wet modal analysis, pipe diameter *D* is 20 m, the distance between the two support piers *L* is 200 m, the pier diameter is 16 m, the pier is 30 m high, and the top of the tunnel pipe is placed 30 m underwater.

Dry and wet modals are simulated and conducted, respectively, and the natural vibration frequencies of each mode are listed in Table 2.

Through comparative analysis of dry and wet modes, it can be found from Table 2 that the natural vibration frequencies of wet modes are all smaller than the corresponding values of the structure in air. According to the basic idea of additional mass method, water acts as an additional mass applied to the structure. From the dynamic equation of the structure, it is evident that the greater the additional mass, the lower the natural frequency of the structure, which demonstrates that the structure of numerical simulation is consistent with the theoretical practice.

#### 5.2.2. Effect of Placement Depth on Natural Vibration Characteristics

The range of the placement depth under water, that is, the distance between the top of the tunnel pipe and the water free surface was selected from 20 m to 150 m to explore the variation rule of the natural vibration frequency of the structure with the water depth.

The results of the first ten natural frequencies at different placement depths based on acoustic-structure coupling method are shown in Table 3.

Under the same water depth, the natural vibration frequency of the structure increases gradually as the modal order is higher. On the whole, although the natural vibration frequency of the structure decreases with the increase of water depth, the frequency drop is very little whose amplitude of variation is basically lower than 0.01 Hz per 10 meters of water depth.

It is proved that water depth is not the key factor affecting the natural vibration of the structure, but considering the environmental factors and construction conditions, water depth is still an indispensable factor. When the water depth is shallow, it may cause disturbance to navigation and other aquatic production activities. When the water depth is too deep, it will bring great difficulties to construction and increase the cost of pipe transportation. In addition, the environment of high water pressure will reduce the stability of the structure. Therefore, the depth of placement should be determined after a comprehensive evaluation of economy and safety based on the actual sea conditions.

#### 5.2.3. Effect of Width-Span Ratio on Natural Vibration Characteristics

**Figure 9:**
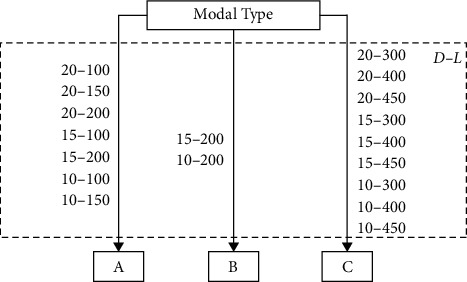
Vibration mode classification.

The single-span was set to 100 m∼450 m, and three sections with diameters of 20 m, 15 m, and 10 m were adopted, while the corresponding pier body was 16 m, 12 m, and 8 m in diameter. The top of tunnel pipe body is 30 m underwater and the pier is 30 m high.  Figure 9 and Table 4 respectively show the classification of each case and the range of width-span ratio with the corresponding description of vibration mode shape for each type.

According to the results of the first three vibration modes among 18 cases, it can be found that, from the perspective of mode shapes, with the span length gradually increasing, the rule of mode has also changed. As the width-span ratio reaches a certain range, the vibration modes can be divided into three types. Type A occurs when the single-span length is short, while the width-span ratio ranges from 0.067 to 0.2, the first-order vibration mode is mainly the bending vibration of pier body with the bottom of pipe totally fixed, while the tunnel pipe body vibrates back and forth in the horizontal direction as a whole. The 2^nd^ and 3^rd^ mode shapes of type A are respectively the horizontal bending vibration and vertical bending of pipe. Then comes the type B as the single-span increases to about 200 m, while the width-span ratio fluctuates between 0.05 and 0.075. Type B is almost like a transitional form presented and the first-order vibration mode turns to be the first-order bending of the pipe body in the horizontal direction, while the 2^nd^ vibration mode is the bending of the pier with the pipe body vibrating back and forth horizontally as a whole, and the 3^rd^ mode shape is the same as Type A. When the span length increases further, the width-span ratio is within a small range from 0.022 to 0.05, the first-order vibration mode is mainly the first-order horizontal bending of the tunnel pipe, with piers torsion, and the 2^nd^ mode shape is vertical bending vibration of pipe, whereas the 3^rd^ vibration mode shape is totally different, where the second-order horizontal bending of pipe body comes up.

From the perspective of natural frequencies, the frequency results of 18 cases are listed in Tables 5–7. With same span length, the natural frequencies increase as modal order goes up. Within the variation range of single-span length from 100 m to 450 m, the width-span ratio of 20 m pipe body varies from 0.2 to 0.22, while the corresponding ratio, for a pipe with a diameter of 15 m and 10 m, ranges respectively from 0.15 to 0.033 and from 0.10 to 0.022.

**Figure 10:**
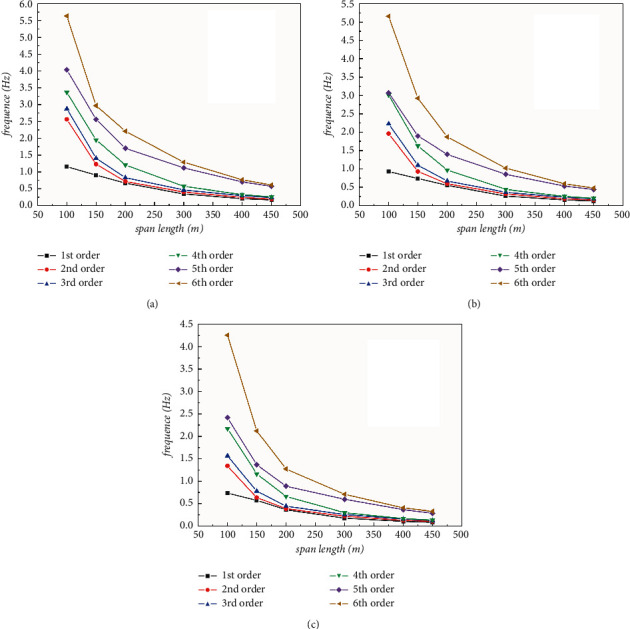
Line chart of natural vibration frequency-span with different pipe diameters: (a) *D* = 20 m; (b) *D* = 15 m; (c) *D* = 10 m.

As is shown from Tables 5–7 and Figure 10 that the natural frequency decreases gradually with the increase of the span length, and the rate of the decrease gradually slows down, when the single-span length increases to 300 m, the first three frequencies of the structure basically tend to coincide, with a small increase per each mode.

**Figure 11:**
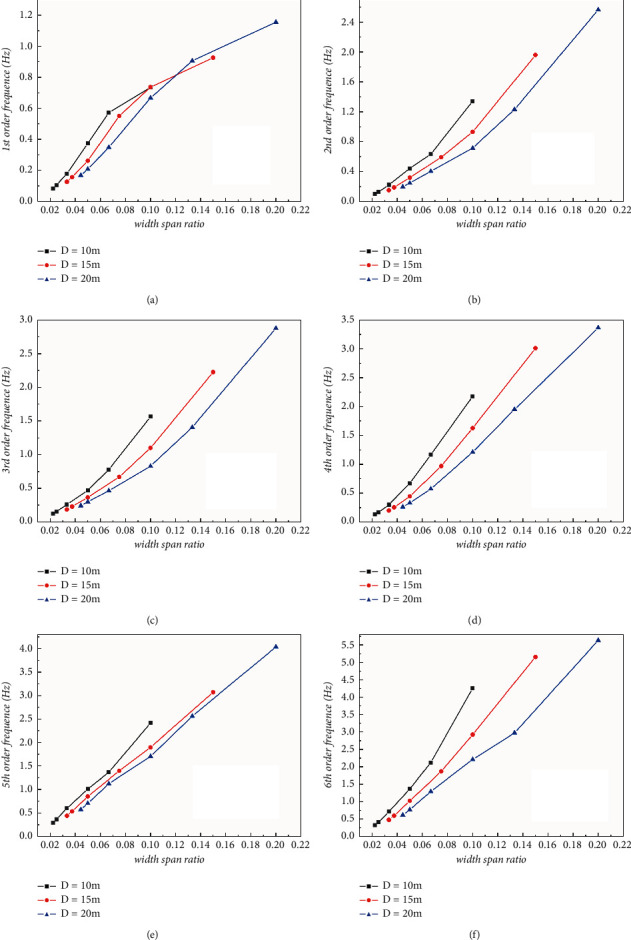
Line chart of natural frequency-width-span ratio in each mode: (a) 1^st^ order; (b) 2^nd^ order; (c) 3^rd^ order; (d) 4^th^ order; (e) 5^th^ order; (f) 6^th^ order.

According to the modal order, the variation diagram of natural vibration frequency with width-span ratio was drawn at the same order, as shown in Figure 11. It can be found that there is a strong positive linear relationship between the natural frequency and the width-span ratio of the typical structure, and the vibration frequency increases gradually as the ratio increases in each mode. Besides, under the same width-span ratio, the natural vibration frequency decreases with the increase of pipe diameter; however, while under the same span length, the natural vibration frequency of the structure increases with the increase of pipe diameter, which fully indicates that the span length has a stronger influence than the cross section size of tunnel pile body on the natural vibration frequency of the assumed structure.

It is a safer design to reduce the span length and avoid the fatigue or damage of the structure due to vortex-induced vibration resonance, which can also improve the stability of the structure. Nevertheless, surplus supporting piers can lead to a substantial rise in the cost of construction. Therefore, the proper structure size should be selected according to the actual water area characteristics and the principle of safety and economy.

### 5.3. Dynamic Response Analysis

#### 5.3.1. Effects of Different Flow Rates

In the studies of the dynamic response of the structure at different flow rates, a circular section with a diameter of 10 m (*D* = 10 m) was selected for the pipe cross section, and the single-span length between the two piers was 200 m (*L* = 200 m), while the calculated water area was 400 m × 300 m × 60 m according to the set above. The study subjects about dynamic response of the structure mainly include the displacement response of the pipe top in all directions and the bending moment as well as the shear force at the bottom of the pier; moreover, the stress, at the junction between the pipe body and the pier column, is also an important object that needs to be monitored.  Table 8 shows the dynamic response results of the structure at different flow rates in the range from 0.5 m/s to 4 m/s.

In Table 8, *Z*_max_ and *Y*_max_ are the maximum horizontal displacement and the maximum vertical displacement of pipe top, Moment_max_ and Shear_max_ are the maximum bending moment and the maximum shear of pier bottom, and Stress_max_ is the maximum stress at the junction between the pipe and pier.

**Figure 12:**
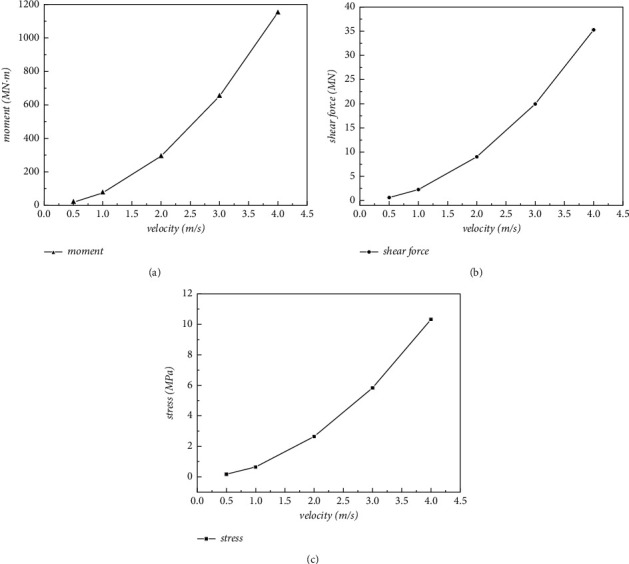
Line chart of dynamic response at different flow rates. (a) Maximum moment of pier bottom Moment_max_. (b) Maximum shear of pier bottom Shear_max_. (c) Maximum stress at the junction Stress_max_.

The main bending moment at the bottom of the pier is axial around the pipe body, showing that the bending of pier bodies occur in the plane where the water cross section is located (YZ plane). As for the shear force of pier bottom mainly acts on the section along the counter-current direction (*Z*-axis negative direction). The numerical results of the maximum bending moment and the maximum shear force at the bottom of the pier, as well as the maximum stress at the junction of the pipe body and the pier body changing with the flow rate, are plotted in Figure 12. As is depicted that with the increase of the flow rate, the bending moment and shear force at the bottom of the pier gradually increased, so as the stress at the junction, and the increase amplitude was nonlinear, showing a quadratic increasing trend.

**Figure 13:**
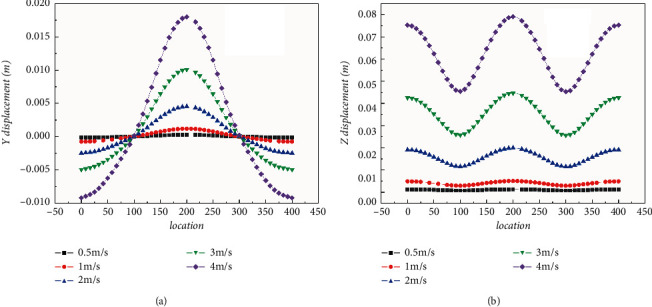
Axial displacement of the top of the pipe. (a) Vertical displacement. (b) Horizontal displacement.

Under the action of ocean current load, the structure displacements occurred in both the downstream direction and the transverse direction, and the maximum displacements occur at the top of the tunnel pipe section, thus the top part of the pipe was set as the monitored path.  Figure 13 shows the vertical and horizontal displacement curves of the path along the longitudinal axis. It can be seen from the figure that the displacement of the pipe body is larger along the ocean current direction than the displacement in the cross current direction. In the vertical direction, the displacements of adjacent spans are in the opposite direction, and the maximum displacement is achieved at the middle of the span, while there is nearly no vertical displacement at the pier support. In the horizontal direction, the horizontal displacement of adjacent spans is in the same direction, and the maximum displacement is achieved at the middle of the span as well, while the minimum displacement is achieved at the pier support. With the increase of flow velocity, the displacement at each point along the pipe also gradually increases, with presenting a quadratic growth trend.

#### 5.3.2. Effects of Different Span Lengths

A circular section with a diameter of 10 m (*D* = 10 m) is selected in the tunnel pipe section, and the single-span length *L* between the two piers is 100 m, 200 m, 300 m, 400 m, and 450 m.  Table 9 shows the dynamic response results of the structure within the span range of 100∼450 m.

**Figure 14:**
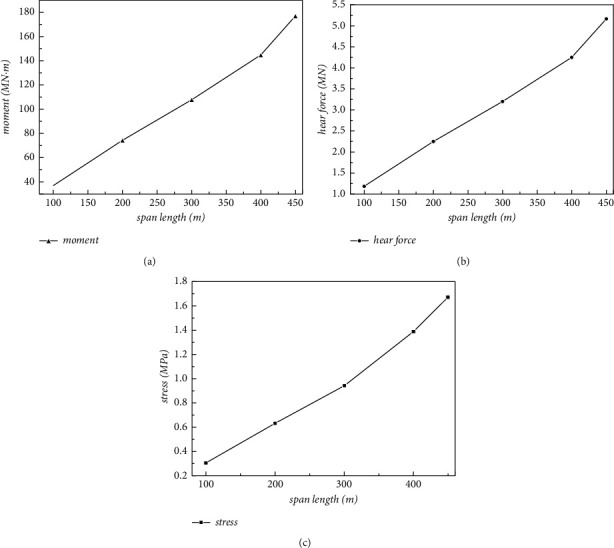
Line chart of dynamic response under different spans. (a) Maximum moment of pier bottom Moment_max_. (b) Maximum shear of pier bottom Shear_max_. (c) Maximum stress at the junction Stress_max_.

Figure 14 shows the numerical results of the maximum bending moment and shear force at the bottom of the pier, as well as the stress at the junction between the pipe body and the pier with the single-span length changing. It can be found that the maximum of the bending moment and shear force at the bottom of the pier gradually increase with the span length increasing, which shows a linear growth trend.

**Figure 15:**
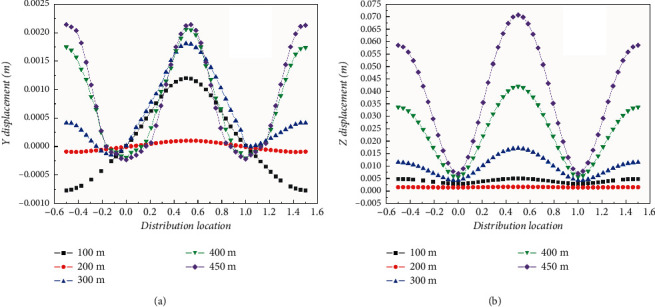
Axial displacement of the top of the pipe. (a) Vertical displacement. (b) Horizontal displacement.

The tunnel pipe top along the axis was set as the monitoring path; Figure 15 shows the axial distribution curves of vertical and horizontal displacements of pipe top under different single-span lengths. It can be found that, in the horizontal direction (*Z* direction), adjacent pipe segments still maintain the same displacement along the flow direction, and the maximum displacement occurs at the middle of the span, while the minimum displacement is located at the pier top support. With the increase of the span length, the horizontal displacement at each point gradually increases.

In the vertical direction (*Y* direction), if the span exceeds a certain range, the displacement response will change. When the single-span length is less than 300 m, the vertical displacements of adjacent spans are reversed, while when the single-span length is longer than 300 m, the vertical displacements of adjacent spans are in the same direction, and the maximum displacements are achieved at the middle of the span, while the vertical displacements at the pier top support are almost zero. With the increase of span length, the maximum of vertical displacement increases gradually.

## 6. Conclusion

A new underwater traffic structure, named as deep water highway tunnel (DWHT), is proposed by comprehensively comparing existing different traffic modes across broad waters. DWHT is a new and competitive solution to the challenge of crossing wide water areas.

In this paper, a simplified numerical simulation model of DWHT (half span + one span + half span) is proposed firstly. Then, the characteristics of flow field around the circular cross section of the underwater tunnel are studied. In addition, dynamic characteristics and dynamic response of the typical structure of DWHT under the action of ocean current are explored. It is found that ocean current velocity and single-span length of tunnel body are the most important influencing factors. Specific conclusions are as follows:

1. Although the variation of flow velocity has little effect on the distribution of circumferential pressure around the circular cross section, the increase of flow velocity can significantly increase the circumferential pressure, as well as the lift force and drag force. However, the fluctuating lift coefficient and steady-state drag coefficient decrease as velocity increases. At the same time, with the increase of the flow velocity, the vortex discharge period gradually decreases, and the Strouhal number fluctuates within a relatively stable range. The Strouhal number of the circular section fluctuates between 0.34 and 0.39.
2. Water can increase the additional mass and reduce the natural vibration frequency of the structure in wet modal. The water depth has unconsidered effect on the natural vibration frequency while the tunnel structure is completely submerged in water, and the natural vibration frequency decreases slightly with the increase of water depth.
3. When the pipe span exceeds a certain range, the vibration mode shape at the same order will change. With the increase of single-span length, the natural vibration frequency of the structure decreases gradually. While the span length exceeds 200 m (the corresponding diameter is 10–20 m), the first-order vibration mode changes from pier bending to pipe horizontal bending.
4. The dynamic responses of the structure have shown the quadratic increasing trend with the increase of flow velocity, including the bending moment, the shear at pier bottom, and the stress at the junction between the pipe body and the pier, as well as the displacements. Besides, the dynamic response results increase linearly with the span length.
5. The maximum displacement of the structure is located at the middle of the top span of the pipe. When the single-span length is short, the vertical displacement of adjacent spans is opposite and the horizontal displacement is in the same direction. When the single-span length is relatively longer, the vertical and horizontal displacements of adjacent spans are both in the same direction.

There still are some limitations in this research, for instance, the internal force between pier body and tube body is not considered, or the variation characteristics of three-dimensional flow field along the axial direction of the tube body are not emphasized, and the quantitative law of the influence of span length (or width-span ratio) on the natural vibration characteristics needs to be further explored.

## Figures and Tables

**Table 1:** Lift and drag coefficients at different flow rates.

Velocity (m/s)	Steady-state lift coefficient	Fluctuating lift coefficient	Steady-state drag coefficient	Fluctuating drag coefficient	Vortex period (s)	Strouhal number
0.5	0.012	0.241	0.358	0.009	58.11	0.3454
1	0.015	0.225	0.296	0.011	25.13	0.3979
2	0.012	0.193	0.278	0.008	13.07	0.3829
3	0.014	0.187	0.255	0.012	9.31	0.358
4	0.009	0.172	0.236	0.015	6.50	0.3845

**Table 2:** Natural frequencies of dry and wet modals.

Order	Dry modal frequencies, *f*_*d*_/*Hz*	Wet modal frequencies, *f*_*d*_/*Hz*	(*f*_*d*_ − *f*_*a*_)/*f*_*d*_
1	1.4468	0.66516	0.540
2	1.5081	0.71504	0.526
3	1.7819	0.82936	0.535
4	2.6011	1.2089	0.535
5	3.5976	1.7013	0.527
6	4.0424	2.2095	0.453
7	5.4398	2.1430	0.606
8	5.8327	2.6401	0.547
9	6.9219	2.8184	0.593
10	6.9655	3.4567	0.504

**Table 3:** The first ten natural frequencies at different water depths (Hz).

Order	Depth of tunnel pipe roof (m)							
	20	30	40	50	60	80	100	150
1	0.66584	0.66516	0.66263	0.66075	0.65984	0.65692	0.65356	0.64704
2	0.71406	0.71504	0.71321	0.71278	0.7125	0.71134	0.7095	0.70527
3	0.82964	0.82936	0.82833	0.82744	0.82767	0.82601	0.82398	0.81834
4	1.2091	1.2089	1.2077	1.207	1.2069	1.2054	1.2027	1.1956
5	1.7021	1.7013	1.70	1.6979	1.6962	1.693	1.6875	1.6758
6	2.21	2.2095	2.2073	2.2069	2.2073	2.2054	2.2039	2.1988
7	2.1458	2.143	2.1411	2.1387	2.1371	2.1351	2.1326	2.1294
8	2.6414	2.6401	2.6384	2.6369	2.6359	2.6326	2.6265	2.6115
9	2.8153	2.8184	2.8134	2.8116	2.8108	2.807	2.8015	2.7884
10	3.4578	3.4567	3.4526	3.4513	3.4525	3.4493	3.4456	3.4279

**Table 4:** Modes with different width-span ratios.

Type	Width-span ratio	Mode shape	
A	0.067∼0.2	1^st^	Bending of pier body
		2^nd^	Horizontal bending of pipe
		3^rd^	Vertical bending of pipe

B	0.05∼0.075	1^st^	Horizontal bending of pipe
		2^nd^	Bending of pier body
		3^rd^	Vertical bent of pipe

C	0.022∼0.05	1^st^	Horizontal bending of pipe
		2^nd^	Vertical bending of pipe
		3^rd^	Second-order horizontal bending of pipe

**Table 5:** Natural vibration frequencies of structures with different span lengths (*D* = 20 m).

Diameter (m)	Span length (m)	Width-span ratio	Frequency (Hz)					
			1^st^	2^nd^	3^rd^	4^th^	5^th^	6^th^
20	100	0.200	1.1554	2.5646	2.8787	3.3667	4.0364	5.6344
	150	0.133	0.90607	1.2315	1.4076	1.9517	2.5616	2.972
	200	0.100	0.66682	0.71533	0.8294	1.209	1.7053	2.2106
	300	0.067	0.34852	0.40492	0.46432	0.57254	1.1229	1.2915
	400	0.050	0.2076	0.24639	0.29847	0.32926	0.70337	0.76766
	450	0.044	0.16769	0.19682	0.24235	0.26155	0.57159	0.61213

**Table 6:** Natural vibration frequencies of structures with different span lengths (*D* = 15 m).

Diameter (m)	Span length (m)	Width-span ratio	Frequency (Hz)					
			1^st^	2^nd^	3^rd^	4^th^	5^th^	6^th^
15	100	0.15	0.92597	1.9591	2.2269	3.0122	3.072	5.1569
	150	0.10	0.73692	0.9308	1.0998	1.6272	1.898	2.9244
	200	0.075	0.55022	0.59316	0.67017	0.96852	1.3962	1.8702
	300	0.050	0.26181	0.31893	0.36445	0.44276	0.85128	1.0218
	400	0.0375	0.1553	0.18946	0.22918	0.25303	0.5329	0.5951
	450	0.033	0.12601	0.15151	0.18604	0.20011	0.4363	0.47561

**Table 7:** Natural vibration frequencies of structures with different span lengths (*D* = 0 m).

Diameter (m)	Span length (m)	Width-span ratio	Frequency (Hz)					
			1^st^	2^nd^	3^rd^	4^th^	5^th^	6^th^
10	100	0.100	0.73518	1.3421	1.5647	2.1748	2.4161	4.2544
	150	0.067	0.57205	0.63435	0.77965	1.1656	1.3685	2.1223
	200	0.05	0.36461	0.3882	0.44204	0.66155	0.8898	1.2757
	300	0.033	0.17839	0.223	0.25877	0.3004	0.59778	0.70932
	400	0.025	0.10585	0.12946	0.15788	0.1691	0.36622	0.40606
	450	0.022	0.083829	0.10547	0.12612	0.13562	0.28867	0.32777

**Table 8:** Dynamic response results of structures at different flow rates.

Velocity (m/s)	0.5	1	2	3	4
*Z*_max_ (mm)	1.258	4.979	19.91	44.62	79.03
*Y*_max_ (mm)	0.415	1.214	4.549	10.10	26.69
Moment_max__(MN·m)_	18.66	73.617	292.24	651.34	1152.1
Shear_max__(MN)_	0.569	2.241	8.930	19.918	35.523
Stress_max_ (MPa)	0.165	0.632	2.615	5.827	10.306

**Table 9:** Dynamic response results of structures with different single-span lengths.

Span (m)	100	200	300	400	450
*Z*_max_ (mm)	1.626	4.979	17.146	42.004	70.667
*Y*_max_ (mm)	0.095	1.214	1.741	2.059	2.12
Moment_max_ (MN·m)	37.14	73.62	107.45	144.20	176.32
Shear_max_ (MN)	1.187	2.241	3.205	4.249	5.163
Stress_max_ (MPa)	0.302	0.632	0.942	1.388	1.671

## Data Availability

The experimental data used to support the findings of this study are available from the corresponding author upon request.
